# Screening candidate genes for fruit size based on QTL-seq in Chinese jujube

**DOI:** 10.3389/fpls.2024.1361771

**Published:** 2024-04-02

**Authors:** Yiling Pan, Yujia Luo, Jingkai Bao, Cuiyun Wu, Jiurui Wang, Mengjun Liu, Fenfen Yan

**Affiliations:** ^1^ College of Horticulture and Forestry, Tarim University/The National-Local Joint Engineering Laboratory of High Efficiency and Superior-Quality Cultivation and Fruit Deep Processing Technology on Characteristic Fruit Trees, Xinjiang, Alar, China; ^2^ College of Forestry, Hebei Agricultural University, Baoding, China; ^3^ College of Horticulture, Hebei Agricultural University, Baoding, China; ^4^ Xinjiang Production & Construction Corps Key Laboratory of Protection and Utilization of Biological Resources in Tarim Basin, Xinjiang, Alar, China

**Keywords:** candidate genes, fruit size, genetic variation analysis, QTL-seq, *Ziziphus jujuba* Mill.

## Abstract

**Introduction:**

Fruit size is an important economic trait affecting jujube fruit quality, which has always been the focus of marker-assisted breeding of jujube traits. However, despite a large number of studies have been carried out, the mechanism and key genes regulating jujube fruit size are mostly unknown.

**Methods:**

In this study, we used a new analysis method Quantitative Trait Loci sequencing (QTL-seq) (bulked segregant analysis) to screen the parents ‘Yuhong’ and ‘Jiaocheng 5’ with significant phenotypic differences and mixed offspring group with extreme traits of large fruit and small fruit, respectively, and, then, DNA mixed pool sequencing was carried out to further shortening the QTL candidate interval for fruit size trait and excavated candidate genes for controlling fruit size.

**Results:**

The candidate intervals related to jujube fruit size were mainly located on chromosomes 1, 5, and 10, and the frequency of chromosome 1 was the highest. Based on the QTL-seq results, the annotation results of ANNOVAR were extracted from 424 SNPs (single-nucleotide polymorphisms) and 164 InDels (insertion-deletion), from which 40 candidate genes were selected, and 37 annotated candidate genes were found in the jujube genome. Four genes (*LOC107428904*, *LOC107415626*, *LOC125420708*, and *LOC107418290*) that are associated with fruit size growth and development were identified by functional annotation of the genes in NCBI (National Center for Biotechnology Information). The genes can provide a basis for further exploration and identification on genes regulating jujube fruit size.

**Discussion:**

In summary, the data obtained in this study revealed that QTL intervals and candidate genes for fruit size at the genomic level provide valuable resources for future functional studies and jujube breeding.

## 1 Introduction

Jujube (*Ziziphus jujuba* Mill.), originated in China, is the largest dried fruit and the seventh largest fruit tree species in China and is also known as one of the traditional “five fruits” in China (Liu et al., 2015). Jujube fruit is rich in nutrition, which can be eaten fresh or dried, and often used as a raw material for Chinese herbal medicines and listed as a medicinal fruit by the government (Bi et al., 2010; Guo et al., 2012). At present, there are more than 900 jujube germplasm resources reported in China, and the trait variation is very rich (Liu and Wang, 2019; Liu MJ et al., 2020). Due to the small flowers of jujube, it is difficult to remove the emasculation. With the extremely low fruit setting rate, the difficulty of traditional artificial pollination operation, and other problems, it was extremely difficult for sexual hybridization of jujube trees. Hence, the hybridization process is very slow, and the genetic research is relatively lagging behind (Yan et al., 2017). The development of modern biotechnology and the wide application of genetic engineering and molecular-assisted selection will greatly accelerate the breeding process (Li et al., 2021; Yuan et al., 2021). Using molecular marker-assisted selection to carry out jujube breeding work, the detection of closely linked genetic markers and genes with the target quantitative traits on the corresponding genome can achieve the transformation from “experiential breeding” to “precise breeding,” promote the selection efficiency of jujube breeding, and realize molecular breeding (Wen et al., 2010).

Fruit size is an important trait affecting fruit quality and has always been a major concern in the breeding process of fruit varieties. Fruit size is a quantitative trait controlled by multiple genes. In addition to the reports on fruit size of the main cultivated fruit trees in China like apple, pear, peach, grape, and citrus (Zhang et al., 2013; Bai et al., 2017; Yan et al., 2017; Zhao et al., 2018; Han et al., 2019; Liu ZH et al., 2020; Wang JJ et al., 2020; Luo, 2021), there are also reports on fruit size of fruit trees such as cherry, kiwifruit, and loquat (Liu, 2016; Zhao, 2018; Zhao et al., 2021). With the construction of jujube hybrid population, some research on fruit size in jujube have been reported. Bao (2022) and Qiu (2021) carried out the construction of genetic map and QTL localization for fruit size traits in two populations with ‘Yuhong’ as the female parent, respectively, and found that there were no distinctive features in the distribution of the QTL loci, and, at the same time, there was no more detailed genetic exploitation of the fruit size traits. Therefore, our study continued to study the fruit size traits of jujube, in order to screen out the candidate genes that control the fruit size of jujube and lay the foundation for the development of molecular-assisted breeding technology in the future.

In recent years, with the construction of multiple hybrid populations of jujube and the gradual popularization of high-throughput sequencing technology (Wang et al., 2019; Yan et al., 2020), it has laid a foundation for the construction of high-density map, fine mapping of fruit size traits, and gene mining of jujube (Xu, 2012; Wang ZT, 2020; Qiu, 2021). However, QTL mapping using genetic maps is usually a labor-intensive, time-consuming, and expensive task to screen DNA markers. Sometimes, due to the low resolution of QTL confidence intervals, the fine mapping of genes related to target traits becomes time-consuming and laborious (Fang and Liu, 2022). The bulked segregant analysis (BSA) method can rapidly identify polymorphic markers linked to target traits, which is an effective and quick method. QTL-seq, combining next-generation sequencing (NGS) and BSA technology, is used for the rapid identification of QTLs. This method speeds up the identification of closely linked markers for important traits and improves the resolution of gene identification and QTL mapping, and, more importantly, QTL-seq can save a lot of time in constructing populations in the aspect of locating quantitative trait loci, so it has become a fast and efficient way to identify gene function or quantitative loci. In summary, QTL-Seq can identify single-nucleotide polymorphism (SNP) loci associated with phenotypes and has been widely used for QTL mapping and functional target genes (Zhang et al., 2020). In the study of quantitative traits of some agronomic crops, DNA mixing pools were constructed by selecting isolated plants with extreme phenotypic differences in parents and offspring for sequencing analysis. For example, QTLs related to seedling vigor and resistance to Phytophthora infestans were successfully identified in rice (Takagi et al., 2013) and cucumber (Liu, 2018) fruit length and pedicel length by QTL-seq, and QTLs and related genes controlling single fruit weight and chamber number were found in tomato (Tiia-Berenguer et al., 2015). In addition, the use of QTL-seq technology has also been reported in fruit trees. A QTL candidate interval with a size of 1.86 Mb was detected in pears to regulate the red/green skin traits of pears (Xue, 2016), and a SNP located in *G8* was found in grapes, which may be closely related to the grape seedless (Wang et al., 2022).

In this study, the fruit size-related traits of 284 offspring of ‘Yuhong’ × ‘Jiaocheng 5’ F1 population were investigated and analyzed; about 30 individuals with extreme large fruited and small fruited, respectively, were selected; and mixed pools were constructed. By the whole-genome sequencing of the parents and selected breeding lines, QTL localization of fruit size traits was carried out through QTL-seq to excavate the genes that potentially regulate fruit size. Our study will promote the molecular-assisted breeding of jujube in the future.

## 2 Materials and methods

### 2.1 Plant material

‘Yuhong’ (JMS2), a typical pollen-free male sterile jujube variety, was used as the female parent. ‘Jiaocheng 5’ (J5), a superior line of jujube, was used as the male parent. In 2015–2016, the net-mask–controlled bee pollination technology was used to control hybridization for 2 consecutive years, and the hybrid offspring lines were obtained in 2017. In 2018, 140 F1 generations and their parents were selected as experimental materials for high grafting in the Qilian Orchard of the Tenth Regiment of the First Division of Xinjiang Production and Construction Corps. The plant spacing was 1 m × 3 m, and the rootstocks were perennial jujubes. In the same year, 144 F1 plants were selected as experimental materials and planted in the seedling base of the Tenth Regiment of the First Division of Xinjiang Production and Construction Corps, with a row spacing of 1 m × 2 m. The fertilizer management level of the two experimental sites was good, and the tree management level was at a medium level.

In 2020 and 2021, the fruit size-related traits of 284 lines of the hybrid population were investigated for 2 consecutive years, and 30 large fruit lines and 30 small fruit lines were selected for the construction of large and small fruit mixed pools. Jujube fruit picking was from 1 September to 20 October in 2020 and 2021. The period to observe the phenotype of fruit ripening was from 10 August to 23 September in 2022, with an interval of 2~4 days (when the whole tree was 90% or more than 90% full red). Fruits with similar size and no pests or diseases were picked from the middle of jujube trees and brought back to the laboratory for pre-treatment. The appearance quality of the fruit was investigated and measured on the same day, and, then, the fruit was mixed (each sample contained more than 30 fruits) and placed at −80°C for later use.

### 2.2 DNA extraction and library sequencing

The leaves of 30 plants of large fruit type and 30 plants of small fruit type selected from the F1 population of ‘JMS2’ × ‘J5’ were placed in cryopreservation tubes and provided to Shanghai Meigi for DNA extraction and detection. DNA concentration was detected by agarose gel electrophoresis. The qualified DNA samples were randomly broken into 350-bp fragments by Covaris pulverizer, and the library was constructed using the Tru Seq Library Construction Kit. The preparation of the whole library was completed by repairing the DNA fragment without end connection, adding ploy a tail, and adding sequencing adapter, and polymerase chain reaction (PCR) was used for purification and amplification. After the library construction was completed, Qubit 2.0 was used for preliminary quantification, and the library was diluted to 1 ng/μL. Then, Agilent 2100 was used to detect the insert size of the library. After the insert size was consistent with the expected, the q-PCR method (the effective concentration of the library > 2 nm) was used to accurately quantify the effective concentration of the library to ensure the quality of the library. After the library test was qualified, different libraries were mixed according to the effective concentration required for Illumina HiSeqTM PE150 sequencing and the amount of target downstream data.

### 2.3 Evaluation of fruit size phenotypes

The fruit size traits of hybrids and their parents were investigated in 2022. The single fruit weight was measured using electronic balance (precision of 0.01 mm) on 30 randomly selected fruits, and the average value was calculated. The fruit length and diameter were measured with a digital Vernier calipers (precision of 0.01 mm). The fruit shape index was the ratio of length to diameter of fruits.

### 2.4 Bioinformatics analysis

The raw data raw reads were obtained and checked using Ilumina Casava version 1.8 [the sequencing error rate is represented by c, and the base mass value of Ilumina HiSeqTM/MiseqTM is represented by Qphred, given by the following formula: Qhred = −10log10(e)]. The original sequencing sequence or original reads obtained after sequencing contain combined, low-quality reads. Filter the raw reads (remove pairs of reads with connectors; remove pairs of paired reads when a single-ended sequencing read contains more than 10% of the proportion of the length of the read; remove pairs of paired reads when a single-ended sequencing read contains more than 50% of the proportion of the length of the read with low-quality bases) to obtain clean reads. The validated sequencing data were then used to compare to the reference genome of jujube by Burrows-Wheeler alignment software (parameter mem 4k 32-M) [National Center for Biotechnology Information (NCBI) download link is https://www.ncbi.nlm.nih.gov/genome/?term=Ziziphus+jujuba], and the comparison results were used to remove duplicates by samtools (parameter rmdup). The comparison content includes the distribution of clean reads on the reference genome, the statistical information such as the comparison efficiency, sequencing depth, genome coverage, and mutation detection of each sample. According to the comparison results, SNP and InDel (insertion-deletion) were detected using the unified genotype module of GATK 3.8 software and filtered using variant filtration (filter parameters for SNP were –cluster Window Size 4, –filter expression “OD< 4.0‖ FS>60.0‖ MO<40.0”, -G filter “GO<20”; for InDel, the filtering parameters were -filter expression “QD<4.0‖ FS>200.0”), and high-quality SNP loci between the test sample and the reference genome were obtained. Finally, the SNP-index method was used to calculate the candidate regions associated with the traits.

The SNP index (Fekih et al., 2013) is to count the number of reads that are the same or dissimilar to the reference genome at a certain base site in the progeny pool and the parent, and calculate the ratio of the number of dissimilar reads to the total number of reads, that is, the SNP index of the base site. To minimize the impact caused by sequencing errors and comparison errors, polymorphic sites in the two daughter pools after SNP-index was calculated were filtered (sites with SNP-index less than 0.3 in both daughters and with SNP depth less than 7 in both daughters were filtered out; sites with missing SNP index in one daughter were filtered out). Then, calculate the difference between the SNP-index of the two offspring, the formula is Δ(SNP-index) = SNP-index (extreme large) − SNP-index (extreme small), the closer the value of Δ(SNP-index) obtained after the subtraction is to 1, which indicates that the greater the degree of association between the marker SNP and the target trait is, then the corresponding window can be used as a candidate region for QTL. Based on the comparison results, the associated genes corresponding to SNPs and InDel within the candidate intervals were annotated with multiple databases [Non-Redundant Protein Database (NR), Swiss-Prot, Gene Ontology (GO), and Kyoto Encyclopedia of Genes and Genomes (KEGG)] for gene function using ANNOVAR software to screen the candidate genes (genes that can cause stop loss or stop gain or that host non-synonymous mutations or variable splice sites are preferred as candidate genes).

### 2.5 Data analysis

We used Microsoft Excel 2019 and SPSS 26.0 to organize and analyze the data obtained from the survey. The plot histograms of corresponding frequency distributions and the normal distributions were standardized by SPSS 26.0. Origin 2022 was used to plot the box line diagrams of the fruit size table values.

## 3 Results

### 3.1 Genetic variation analysis of fruit size

**Figure 1:**
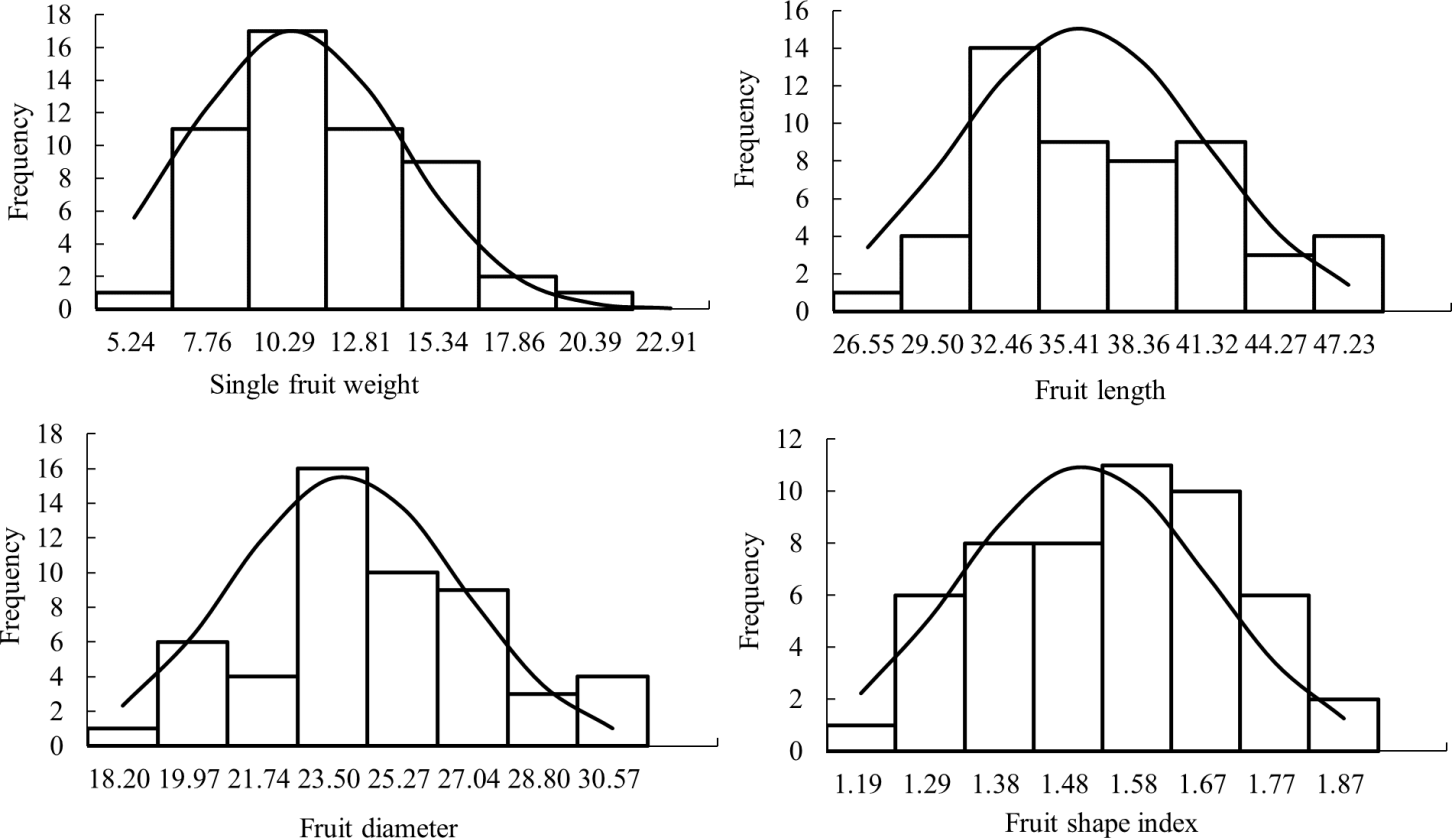
Histogram of frequency distribution of fruit size traits of F1 hybrids in jujube.

The analysis of the frequency distribution histogram of fruit size traits (**Figure 1**) showed that all of them showed continuous variation and conformed to normal or skewed normal distribution, with typical genetic characteristics of quantitative traits being micro-effect polygenes control of quantitative traits. The skewness value of single fruit weight is large, and the normal distribution diagram shows that the single fruit weight has an obvious right deviation distribution. The results were similar to those of the research group in 2020 and 2021 (Bao, 2021), indicating that the inheritance of fruit size traits tended to be stable.

The single fruit weight, fruit length, diameter, and shape index of F1 generation of ‘Yuhong’ × ‘Jiao 5’ were investigated and analyzed in 2022 (**Table 1**). The results showed that the coefficient of variation of fruit size traits ranged from 11.67% to 33.52%, indicating that the fruit size traits of jujube F1 hybrids were widely separated. The coefficient of variation of single fruit weight was the largest, which was 33.52%. In addition, the fruit shape index was the smallest, just 11.67%. The mid-parent heterosis rate of single fruit weight, fruit length, and fruit diameter were negative, and the average value was less than the mid-parent value. Therefore, it was speculated that the single fruit weight, fruit length, and diameter tended to be positive. However, there were ultra-high-parent plants for all fruit size traits in the F1 population. The super high parent rate of fruit shape index was the highest, which was 16.98%, and its traits had obvious heterosis in F1 generation. The single fruit weight was the lowest, which was 7.55% and the heterosis of its traits in F1 generation was not obvious.

**Table 1:** Genetic variation analysis of fruit size traits in the F1 jujube hybrids in 2022.

Traits	JMS2	J5	*V*_MP_	F1									
	Mean			Mean ± *SD*	Min	Max	*CV* (%)	*HH* (%)	*L* (%)	*R*_Hm_ (%)	*Ta* (%)	Kurtosis	Skewness
**Single fruit weight (g)**	11.40	15.79	13.60	10.50 ± 3.52	5.24	22.91	33.52	7.55	73.58	−22.80	77.20	1.87	1.20
**Fruit length (mm)**	35.78	44.72	40.25	35.69 ± 5.31	26.55	47.23	14.87	7.55	54.72	−11.32	88.68	−0.80	0.42
**Fruit diameter (mm)**	24.83	26.82	25.83	23.83 ± 2.89	18.20	30.57	12.12	15.09	67.92	−7.73	92.27	−0.29	0.22
**Fruit shape index**	1.44	1.67	1.55	1.50 ± 0.18	1.19	1.87	11.67	16.98	33.96	−3.31	96.69	−0.85	−0.08

### 3.2 Screening of extreme strains in the F1 population

**Figure 2:**
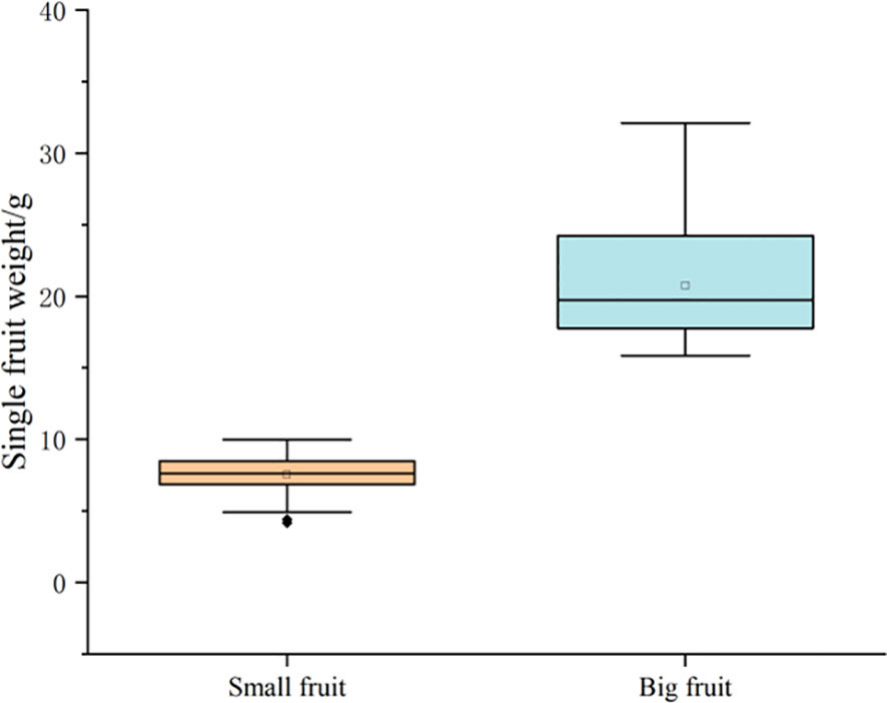
Box line diagram of fruit size table values.

In this experiment, the fruit size traits of the F1 generation population in 2020 and 2021 were investigated for 2 consecutive years. The fruit weight of a single fruit was used as the main screening index, and the vertical and horizontal diameter of fruit was used as auxiliary screening indexes. Combined with the survey results in 2022, the phenotypic values of fruit size of these 60 lines were also analyzed. The results showed that the fruit weight of large fruit ranged from 15.86 g to 32.12 g and that the fruit weight of small fruit ranged from 4.15 g to 9.99 g. The two extreme pools were significantly different in phenotypic traits (**Table 2**, **Figure 2**). Therefore, the expression of the 60 plants screened was not significantly different in the genetic background, which could be used to construct the fruit size BSA sequencing of the library.

**Table 2:** Survey and analysis of large and small fruits from F1 hybrids for 3 years.

Years	Traits	Small fruit		Large fruit	
		Range of variability	Mean ± *SD*	Range of variability	Mean ± *SD*
2020/2021/2022	Single fruit weight/g	4.15–9.99	7.54 ± 1.39	15.86–32.12	20.76 ± 4.04
	Fruit length/mm	19.10–39.12	30.51 ± 4.06	37.47–55.16	43.64 ± 4.52
	Fruit diam/mm	19.23–25.62	22.09 ± 1.85	26.02–35.41	30.06 ± 2.29

### 3.3 Quality control analysis of sequencing raw data

The quality analysis of sequencing data, such as sequencing depth, base content, and coverage, is used to determine whether the data are standard. Therefore, high-throughput sequencing analysis was performed on four samples (large fruit pool, small fruit pool, JMS2, J5) for detection (**Table 3**). The raw data were filtered to obtain a total of 75.36 G clean data, with 6.62 G of clean data for the male parent and 6.76 G for the female parent, as well as 24.74 G of clean data for the large fruit pool and 37.24 G for the small fruit pool. After examining them, it was found that Q20 > 97.7% and Q30 > 93.47%, and the GC content was in the range of 34.58% to 35.5%. It indicates that the amount of sample data is sufficient, the sequencing quality is high, and the GC distribution is normal, which means that the library sequencing is successful and the measured data can be further analyzed.

**Table 3:** Statistical analysis of sample sequencing data evaluation.

Sample	Clean base (bp)	Q20 (%)	Q30 (%)	GC content (%)
Small fruit pond	37,240,231,632	98.35	94.25	34.67
Big fruit pond	24,741,180,842	98.22	94.33	35.3
JMS2 (♀)	6,762,077,180	97.71	93.47	34.58
J5 (♂)	6,620,859,826	97.7	93.47	35.5

Clean bases: The number of filtered bases in bp. Q20 (%) and Q30 (%): Proportion of bases with mass numbers greater than 20 and 30 to the total number of bases. GC content (%): The ratio of the sum of the number of bases G and C to the total number of bases.

### 3.4 Comparative analysis of data after quality control with reference genomes

The data obtained after quality control in the samples were compared with the corresponding winter jujube reference genome (**Table 4**), and the results showed that the comparison rate of the measured samples was between 98.3% and 98.69%, with a high percentage. The average coverage depth of sequencing samples on the reference genome ranged from 14.37× to 82.17×, of which 1× coverage was above 91.89% and 4× coverage was above 84.83%. The results show that the data meet the criteria of mutation detection and analysis and can be used for subsequent mutation detection and correlation analysis.

**Table 4:** Statistical analysis of comparison results with the reference genome.

Sample	Mapped reads	Total reads	Mapping rate (%)	Average depth (×)	Coverage at least 1× (%)	Coverage at least 4× (%)
Small fruit pond	245,734,467	249,167,384	98.62	82.17	97.46	95.82
Big fruit pond	163,730,612	166,566,052	98.3	55.38	95.95	94.05
JMS2 (♀)	44,551,023	45,151,054	98.67	14.42	92.03	86.32
J5 (♂)	43,631,130	44,209,662	98.69	14.37	91.89	84.83

Mapped reads: Number of reads compared to the reference. Mapping rate: Comparison rate, the number of reads compared to the reference genome divided by the number of reads of valid sequencing data. Coverage at least 1×/4×: Reference genome with at least 1/4 base coverage of loci as a proportion of the genome.

### 3.5 Variation detection

Because codon differences arise from differences in bases, including non-synonymous mutations, code-shift mutations, and other mutations, non-synonymous mutations directly cause differences in proteins, whereas InDel brings about changes or loss of gene function. At the same time, structural variations in genes cause changes in function and structure. The sequenced samples were compared with the reference genome of jujube and analyzed for differences at the DNA level using the GATK 3.8 software, and, then, the detected variant sites were annotated using ANNOVAR (**Tables 5**, **6**). According to its annotation statistics, there are 3,552,834 SNPs, of which 98,147 are non-synonymous mutations; and there are 818,788 InDel, of which 3,417 are shifted code mutations.

**Table 5:** Statistics of SNP detection and annotation results.

Category		Number of SNPs
Upstream		191,893
Exon	Stop gain	1,757
	Stop loss	274
	Synonymous	80,145
	Non-synonymous	98,147
Intronic		357,677
Splicing		523
Downstream		180,087
Upstream/downstream		18,197
Intergenic		2,622,790
Transitions (Ts)		2,260,594
Transversions (Tv)		1,292,240
Ts/Tv		1,749
Total		3,552,834

**Table 6:** Statistics of InDel detection and annotation results.

Category		Number of InDels
Upstream		77,639
Exonic	Stop gain	122
	Stop loss	38
	Frameshift deletion	2,060
	Frameshift insertion	1,357
	Non-frameshift deletion	1,552
	Non-frameshift insertion	1,343
Intronic		97,808
Splicing		245
Downstream		58,873
Upstream/downstream		6,987
Intergenic		570,664
Insertion		398,001
Deletion		420,787
Total		818,788

### 3.6 SNP-index association analysis

**Figure 3:**
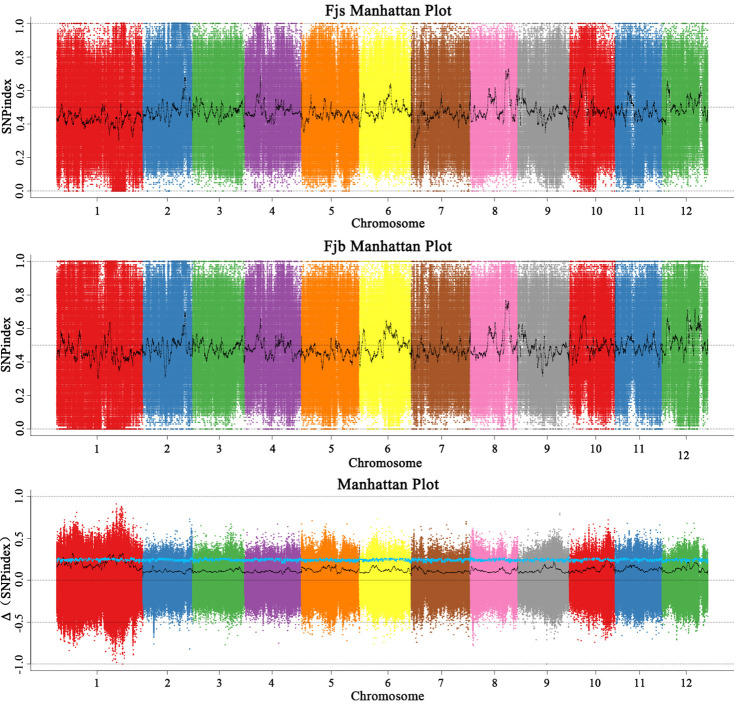
Distribution of SNP-index association values on chromosomes. Fjs denotes the DNA pool of small fruits in the F1 population; Fjb denotes the DNA pool of large fruits in the F1 population.

Before the association analysis, the effect of sequencing and alignment errors was reduced by referring to its corresponding reference genome. The SNP-index of each SNP locus was analyzed and calculated, and the polymorphic loci were screened to obtain 842,847 polymorphic marker loci. According to the SNP-index method, candidate intervals are selected for windows with thresholds greater than 3.0 at a 95% (blue) confidence level. The SNP-index map (**Figure 3**) was drawn by using the sliding window (1 Mb was selected as the window, and 1 kb was selected as the step size) strategy. The chromosome length (Mb) was used as the abscissa, and the SNP-index value (ΔSNP-index) was used as the ordinate. The distribution of SNP-index on chromosomes was analyzed, and 10 chromosome regions related to fruit size were obtained, which were located on chromosomes 1, 5, and 10, respectively. It was mainly concentrated on chromosome 1, and there were nine candidate intervals, which were 3.59 Mb, 1.26 Mb, 2.51 Mb, 2.40 Mb, 1.00 Mb, 1.25 Mb, 5.76 Mb, 5.21 Mb, and 1.81 Mb, respectively. The minimum interval was 1.00 Mb, which contained 16 genes. There was an interval on chromosome 5 with a size of 1.01 Mb, and no associated gene was found. There was an interval on chromosome 10 with a size of 2.85 Mb. The results of the SNP-index algorithm showed that the total length of the final obtained region was 28.64 Mb, containing a total of 2,222 genes.

### 3.7 Correlation analysis of the InDel-index

Before association analysis, the effects of sequencing and alignment errors were reduced by referring to their corresponding reference genomes. After analyzing and calculating the InDel-index of each InDel locus, the obtained polymorphic loci were filtered to obtain 475,073 polymorphic marker loci.

**Figure 4:**
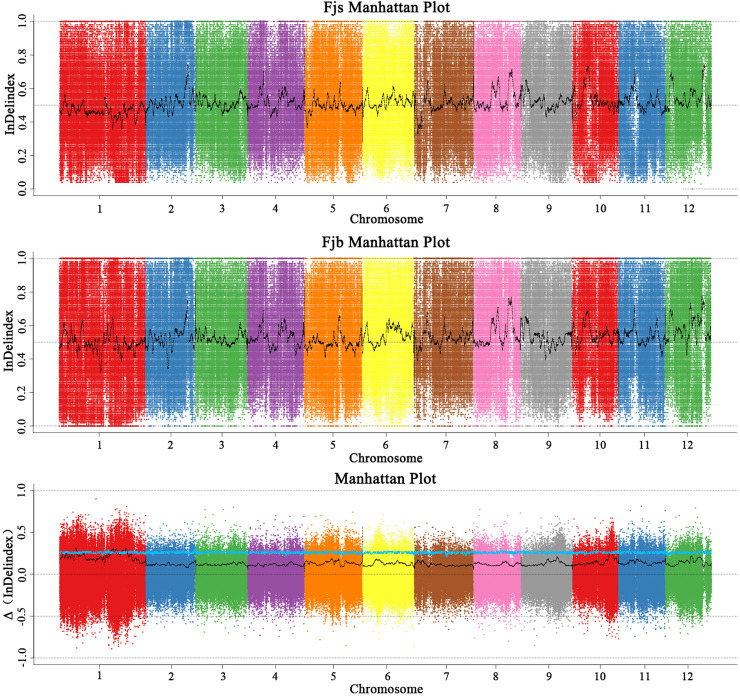
Distribution of InDel-index association values on chromosomes. Fjs denotes the DNA pool of small fruits in the F1 population; Fjb denotes the DNA pool of large fruits in the F1 population.

According to the InDel-index method, candidate intervals are selected for windows with a threshold greater than 3.0 at a 95% (blue) confidence level. InDel-index (**Figure 4**) was drawn by sliding window strategy (1 Mb as window and 1 kb as step), chromosome length (Mb) was used as abscissa, and InDel-index (ΔInDel-index) was used as ordinate. The distribution map of InDel-index on chromosomes was analyzed, and six chromosome regions related to fruit size were obtained, which were located on chromosomes 1, 5, and 10, respectively. It was mainly concentrated on chromosome 1, and there were six candidate intervals, which were 1.06 Mb, 1.09 Mb, 2.53 Mb, 2.20 Mb, 1.00 Mb, and 10.57 Mb, respectively. The minimum interval was 1.06 Mb, containing 109 genes. There was an interval on chromosome 5 with a size of 1.00 Mb, and no associated gene was found. On chromosome 10, there is an interval with a size of 2.12 Mb. The results of the InDel-index algorithm showed that the total length of the obtained region was 20.56 Mb, containing a total of 1,578 genes.

### 3.8 All-index correlation analysis

**Figure 5:**
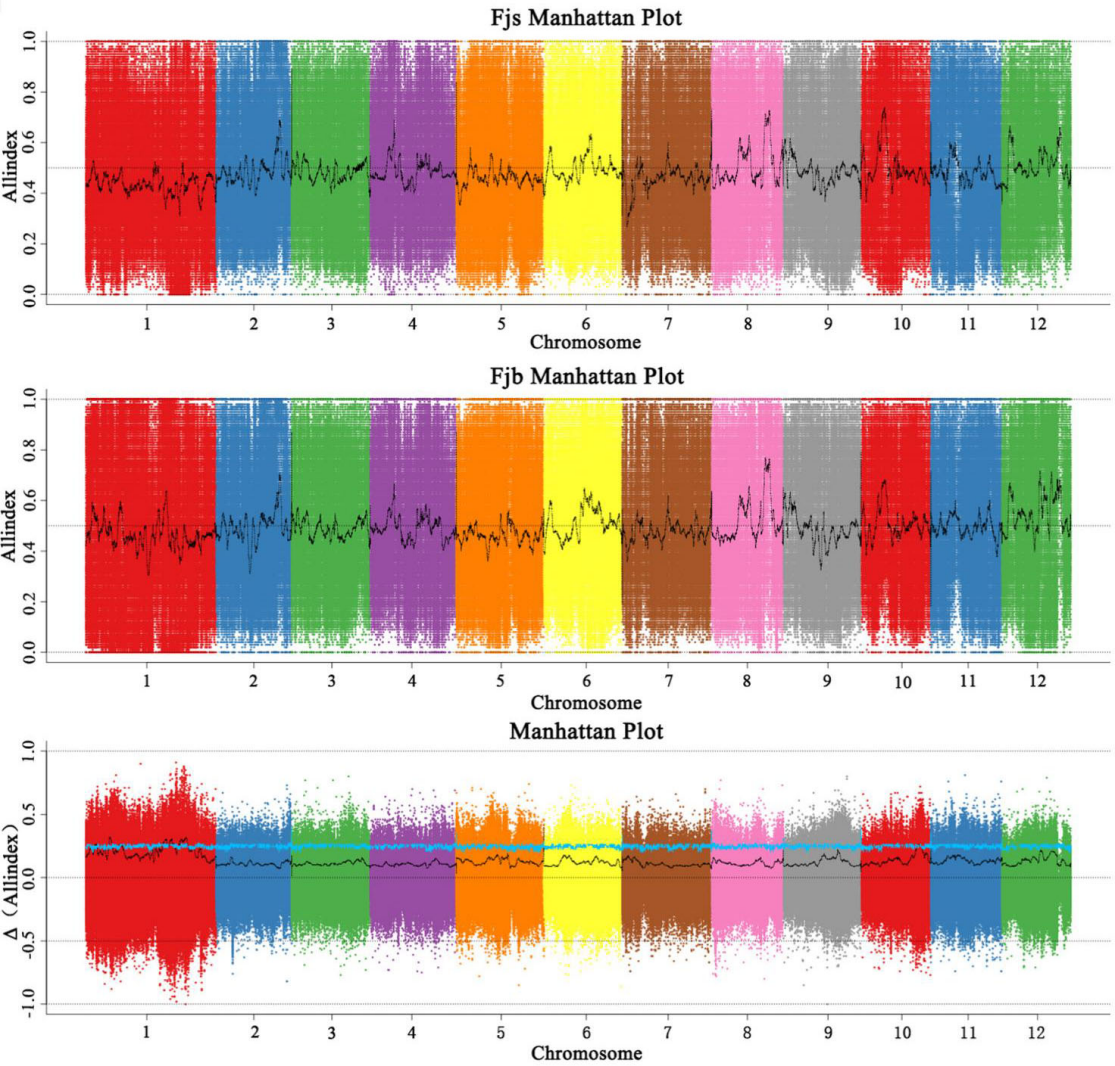
Distribution of All-index association values on chromosomes. Fjs denotes the DNA pool of small fruits in the F1 population; Fjb denotes the DNA pool of large fruits in the F1 population.

The SNP-index and InDel-index were combined according to the All-index method, and candidate intervals were selected for windows with a threshold greater than 3.0 at a 95% (blue) confidence level. The sliding window strategy (1 Mb as the window and 1 kb as the step size) was used to draw the All-index map (**Figure 5**). The chromosome length (Mb) was on the horizontal axis, and the All-index (ΔAll-index) value was on the vertical axis. The distribution map of All-index on chromosomes was analyzed, and a chromosome region related to fruit size was obtained, which was located in the region of 30.72 Mb to ~32.23 Mb on chromosome 1. The interval size was 1.51 Mb, containing a total of 74 genes.

### 3.9 QTL-seq based candidate gene screening

In order not to ignore the role of minor QTLs, candidate SNPs and InDel loci through the whole genome were selected, and candidate genes according to the ANNOVAR annotation results extracted from candidate loci were further screened. As shown in **Table 7**, in the 424 SNP polymorphic marker loci selected, 25 genes were prioritized as candidate genes according to the annotation results of ANNOVAR, of which 22 candidate genes could be found in the jujube genome. It can be seen from **Table 8** that, after 164 InDel polymorphic marker loci were selected, according to the annotation results of ANNOVAR, 15 genes were preferentially selected as candidate genes, of which 14 candidate genes could be found on the jujube genome. Annotation, candidate genes are mainly distributed on chromosome 1.

**Table 7:** Gene annotation of candidate SNP loci.

Trans ID	Variant	Chromosome no.	Pos.	Ref.	Alt.	Description
*LOC107428904*	Nonsynonymous	1	34,789,744	G	A	G-type lectin S-receptor–like serine/threonine-protein kinase CES101
*LOC125419419*	Nonsynonymous	1	37,809,384	T	C	Autophagy-related protein 9–like
*LOC107429678*	Upstream	1	2,119,756	A	T	60S ribosomal protein L7-2–like
*LOC107410261*	Upstream	1	2,800,834	T	A	Stress response protein nst1
*LOC107410630*	Upstream	1	4,282,464	C	G	Protein LIGHT-DEPENDENT SHORT HYPOCOTYLS 10–like
*LOC107415626*	Upstream	1	7,729,619	G	A	Probable 26S proteasome regulatory subunit 10B
*LOC107415838*	Upstream	1	9,506,661	C	A	Oligopeptide transporter 3
*LOC107416052*	Upstream	1	9,809,056	T	A	Probable galactinol-sucrose galactosyltransferase 2
*LOC107416646*	Upstream	1	10,062,515	C	T	L10-interacting MYB domain-containing protein–like
*LOC112492633*	Upstream	1	12,282,277	C	T	Uncharacterized LOC11249263
*LOC112492633*	Upstream	1	12,282,344	A	T	Uncharacterized LOC11249263
*LOC107427361*	Upstream	1	31,195,702	T	C	60S ribosomal protein L39
*LOC107427319*	Downstream	1	31,195,702	T	C	DEAD-box ATP-dependent RNA helicase 57
*LOC107427897*	Upstream	1	32,085,198	T	G	Uncharacterized LOC107427897
*LOC125421212*	Upstream	1	32,521,722	G	A	Triphosphate tunnel metalloenzyme 3–like
*LOC125421212*	Upstream	1	32,521,754	T	C	Triphosphate tunnel metalloenzyme 3–like
*LOC107434928*	Upstream	1	33,891,909	C	A	Methyl-CpG-binding domain-containing protein 11–like
*LOC107434812*	Upstream	1	34,246,119	A	G	Pentatricopeptide repeat-containing protein At5g66520–like
*LOC125420706*	Upstream	1	36,463,835	T	C	Secreted RxLR effector protein 161–like
*LOC125420706*	Upstream	1	36,464,517	T	C	Secreted RxLR effector protein 161–like
*LOC125420706*	Upstream	1	36,464,535	C	T	Secreted RxLR effector protein 161–like
*LOC125420706*	Upstream	1	36,464,552	C	T	Secreted RxLR effector protein 161–like
*LOC125420706*	Upstream	1	36,464,609	C	T	Secreted RxLR effector protein 161–like
*LOC125420706*	Upstream	1	36,464,625	G	A	Secreted RxLR effector protein 161–like
*LOC125420706*	Upstream	1	36,464,640	C	T	Secreted RxLR effector protein 161–like
*LOC125420708*	Upstream	1	36,466,668	T	C	G-type lectin S-receptor–like serine/threonine-protein kinase At4g27290
*LOC125419419*	Upstream	1	37,809,400	T	C	Autophagy-related protein 9–like
*LOC107431296*	Downstream	1	37,809,400	T	C	Oxysterol-binding protein-related protein 1C–like
*LOC125419419*	Upstream	1	37,809,925	A	G	Autophagy-related protein 9–like
*LOC107431296*	Downstream	1	37,809,925	A	G	Oxysterol-binding protein-related protein 1C–like
*LOC107435351*	Upstream	1	46,645,449	G	A	Protein OXIDATIVE STRESS 3 LIKE 2–like
*LOC107429729*	Upstream	10	24,836,424	C	T	Serpin-ZXA–like
*LOC107429866*	Upstream	11	3,879,679	A	G	Beta-adaptin–like protein C

**Table 8:** Gene annotation status of candidate InDel loci.

Trans ID	Variant	Chromosome No.	Pos.	Ref.	Alt.	Description
*LOC107416646*	Upstream	1	10,062,550	–	G	L10-interacting MYB domain-containing protein–like
*LOC107416756*	Upstream	1	10,130,840	ATAAAAAATAAAAGAAAGAAGTTGTAATTAAC	–	Peptidyl-prolyl cis-trans isomerase FKBP16-1
*LOC112492633*	Upstream	1	12,282,567	AAA	–	Uncharacterized LOC112492633
*LOC107419709*	Upstream	1	13,411,848	–	A	Proteasome subunit alpha type-2-A
*LOC107418147*	Upstream	1	32,325,602	–	TTTTG	Cysteine-rich receptor–like protein kinase 44
*LOC107434932*	Upstream	1	33,869,908	ATTTGCATT	–	Glucan endo-1,3-beta-glucosidase 11–like
*LOC107434812*	Upstream	1	34,246,362	C	–	Pentatricopeptide repeat-containing protein At5g66520–like
*LOC107434812*	Upstream	1	34,246,380	C	–	Pentatricopeptide repeat-containing protein At5g66520–like
*LOC107434812*	Upstream	1	34,246,452	A	–	Pentatricopeptide repeat-containing protein At5g66520–like
*LOC107433024*	Upstream	1	39,609,566	–	TATT	Calcium-dependent protein kinase 2
*LOC107433077*	Upstream	1	39,634,711	–	C	Syntaxin-61
*LOC107433077*	Upstream	1	39,634,759	A	–	Syntaxin-61
*LOC107404388*	Upstream	1	45,148,484	TATTTT	–	UDP-glucosyltransferase 29–like
*LOC107418290*	Upstream	5	9,505,773	–	TG	AP-1 complex subunit mu-2–like
*LOC107420246*	Upstream	6	6,409,038	–	TGTG	Abscisic acid 8’-hydroxylase 2
*LOC107421064*	Upstream	6	11,024,545	TCTC	–	UDP-galactose transporter 1
*LOC107423890*	Upstream	8	3,467,453	–	TG	Germin-like protein
*LOC125419526*	Upstream	12	12,839,536	–	TA	Loricrin-like

Transcriptional expression and gene function prediction of 37 annotated genes were performed on the basis of the database on the NCBI website. Four genes that may be related to fruit size growth and development were screened, namely, *LOC107428904*, *LOC107415626*, *LOC125420708*, and *LOC107418290*. Among them, *LOC107428904* and *LOC125420708* regulate plant growth and development and are more abundant in apical meristems located in roots and stems; *LOC107415626* regulates the cell cycle, cell division, chromosome separation, and signal mechanism of plant cells, and is also related to the biosynthesis of plant cell wall/membrane/envelope, and *LOC107418290* mainly regulates the early growth and development of plants and regulates cytokinin to establish meristems for phloem development.

## 4 Discussion

### 4.1 Candidate interval analysis based on QTL-seq technique

In order to search for genes that control quantitative traits, based on the construction of a high-density genetic map of fruit tree hybrid populations, we used QTL-seq technology to analyze candidate intervals, then detected QTL loci with reference to the corresponding fruit tree genomes, and carried out gene mining and functional verification for the studied quantitative traits. Although some achievements have been made in fruit trees (Yuan et al., 2021), this method requires the development and selection of DNA markers for linkage analysis, and QTL analysis is time-consuming and laborious. The advantage of using QTL-seq is that the use of extreme phenotypic individuals in the segregating population has a more consistent genetic background and more accurate and reliable results for specific target traits and does not require DNA molecular markers, which can greatly reduce the candidate region and reduce the time for molecular marker development. Nowadays, the use of QTL-seq technology in crops (Ontoy et al., 2023) and vegetables (Liu, 2018) has been reported, but there are few related reports because it is not easy to establish hybrid segregation populations in fruit trees (Chen et al., 2022). In this study, QTL-seq technology was used to analyze the related loci of jujube fruit size. Through the SNP-index, InDel-index, All-index algorithm, and the corresponding interval association analysis of SNP and InDel, the interval loci regulating fruit size were mainly distributed on chromosome 1. When the team’s previous researchers used genetic mapping to locate QTLs for fruit size traits, the mapping segments that regulate fruit size traits were mainly distributed on chromosome 1 (Bao, 2022), which proved the feasibility of this method and was faster and more economical than using genetic mapping to locate target traits.

### 4.2 Candidate gene analysis based on QTL-seq technology

In this study, candidate SNP and InDel candidate genes were screened on a genome-wide scale. A total of 40 candidate genes were obtained, and 37 annotated candidate genes were found on the jujube genome. The candidate genes were mainly distributed on chromosome 1.

According to the annotation of candidate genes in the NCBI database and the reports of other plants in the relevant literature, four genes were found to be related to growth and development genes. The gene annotation was G-type lectin S-receptor–like serine/threonine-protein kinase CES101; probable 26S proteasome regulatory subunit 10B; G-type lectin S-receptor–like serine/threonine-protein kinase At4g27290; AP-1 complex subunit mu-2–like. The expression of GsSRK protein is usually induced by ABA, salt, and drought stress. In the report by Sun et al. (2013), the expression of *GsSRK* in Arabidopsis thaliana promoted the germination of seeds and the growth of primary roots and rosette leaves in the early stage of salt stress. In the comparison of Arabidopsis thaliana expressing and non-expressing the protein, the plants expressing the protein showed better salt tolerance, increased plant height, and increased yield. Through NCBI database search, it was found that the same possible 26S proteasome regulatory subunit 10B annotated protein was also found on the *LOC122276147* and *LOC122279628* genes of Juglans regia (Mo et al., 2018). It belongs to the SpoVK/Ycf46/Vps4 family and regulates the occurrence of healing tissue. In the AP-1 complex subunit mu-2–like, an annotated protein generally regulates floral meristems by regulating cytokinin, which is more pronounced in *Arabidopsis* flowers. Han et al. (2014) found that the MADS-box transcription factor encoded by the floral isomer gene *AP1* regulates cytokinin homeostasis by directly activating the cytokinin degradation gene *CYTOKININ OXIDASE DEHYDROGENASE3* (*CKX3*) and inhibiting the cytokinin biosynthesis gene *LONELY GUY1* (*LOG1*). In the future, these genes should be functionally verified and their expression in jujube fruits should be observed.

### 4.3 Integrated analysis of traditional QTL localization and QTL-seq techniques

With the rapid development of molecular breeding, some scholars have combined two QTL mapping methods for analysis. Lu et al. (2014) reduced the interval of cucumber flowering gene to 890 kb by genetic mapping and QTL-seq. In the study of rice (Guo, 2019), the QTL interval of alkali tolerance in rice was analyzed by traditional QTL mapping combined with BSA-seq, and the target interval was narrowed to 465 kb on chromosome 2. Among the 65 candidate genes screened, 47 genes were successfully annotated. Yao et al. (2016) performed QTL mapping analysis of rice 1,000-grain weight through multiple environments. After detecting the main-effect QTL that can be stably expressed, the BSA-seq was used to reduce it to a range of 1.47 Mb. In addition, on fruit trees, Jia (2018) used both Map QTL and BSA-seq to comprehensively analyze the QTL loci for malic acid content in fruits, and the results showed that there were four primary effector QTLs were localized, as well as four candidate genes were screened: *MdMYB44*, *MdPP2CH*, *MdSAUR37*, and *MdALMTII*. Therefore, QTL-seq technology can locate major QTL faster and more accurately than other traditional methods, and accelerate the detection of complex traits or mutant QTL. The results of this study can provide reference for precise quantitative trait positioning of jujube trees, improving breeding direction and breeding efficiency, and cross-breeding of jujube trees in future. It also has reference value for other fruit trees.

## 5 Conclusions

In this study, 284 F1 populations of jujube constructed by ‘Yuhong’ × ‘Jiaocheng 5’ were used as test materials. Based on QTL-seq technology, the fruit size of jujube was analyzed. The candidate intervals regulating fruit size were located on chromosomes 1, 5, and 10, mainly distributed on chromosome 1. Using QTL-seq technology, based on the annotation results of ANNOVAR, 40 genes were selected as candidate genes from 424 SNP polymorphic markers and 164 InDel polymorphic markers, respectively, and 37 of them could be found in the jujube genome. According to the data information of the NCBI website, four genes that may be related to the growth and development of fruit size were found, namely, *LOC107428904*, *LOC107415626*, *LOC125420708*, and *LOC107418290*, respectively. Functional verification can be carried out subsequently. The results of this study can provide important application value for hybrid breeding and molecular-assisted breeding of jujube.

## Data availability statement

The data presented in the study are deposited in the NCBI repository, accession number PRJNA1092457 (https://www.ncbi.nlm.nih.gov/bioproject/PRJNA1092457).

## Author contributions

YP: Writing – original draft. YL: Writing – original draft. JB: Writing – original draft, Investigation. CW: Writing – review & editing, Investigation, Conceptualization. JW: Writing – review & editing, Methodology. ML: Writing – review & editing, Methodology. FY: Writing – review & editing, Visualization, Methodology, Investigation, Funding acquisition.
